# Sexual Dimorphism for the Association between Vitamin D and Insulin Resistance in Chinese People

**DOI:** 10.1155/2018/1216370

**Published:** 2018-03-06

**Authors:** Bing Han, Qin Li, Ningjian Wang, Yi Chen, Chunfang Zhu, Yingchao Chen, Fangzhen Xia, Zhen Cang, Meng Lu, Ying Meng, Chi Chen, Yingli Lu

**Affiliations:** Institute and Department of Endocrinology and Metabolism, Shanghai Ninth People's Hospital Affiliated to Shanghai Jiaotong University School of Medicine, Shanghai, China

## Abstract

**Background:**

The relationship between vitamin D and insulin resistance is still controversial. Many factors could influence this relationship. In addition, this relationship in different genders was still unclear.

**Methods:**

A total of 6597 subjects, including 2813 males and 3784 females, were analyzed. The serum levels of 25(OH)D, fasting blood glucose (FBG), fasting insulin, HbA1c, and other metabolic parameters were tested. The waist circumference (WC), weight, and height were also measured. Questionnaires regarding smoking and drinking were collected from these subjects.

**Results:**

Serum 25(OH)D was categorized into quartiles. Increasing 25(OH)D levels were associated with reduced trend of homeostasis model assessment of insulin resistance (HOMA-IR) in both males and females. Pearson's correlation indicated 25(OH)D level was inversely associated with the HOMA-IR for male subjects (*r* = −0.055, *P* = 0.028) but not for female subjects (*r* = −0.005, *P* = 0.798). Age, triglyceride (TG), and low-density lipoprotein (LDL) were associated with the vitamin D levels in males and females. In regression analysis, serum 25(OH)D concentration was significantly associated with HOMA-IR only in overweight males.

**Conclusion:**

We found an inverse association between 25(OH)D and HOMA-IR in Chinese overweight males. Vitamin D supplementation might be beneficial in this population. However, further clinical trials are needed to confirm this association.

## 1. Introduction

As a major noncommunicable disease, diabetes is becoming more and more prevalent in China. According to the 2010 American Diabetes Association (ADA) criteria for diabetes, the estimated prevalence is 11.6% for diabetes and 50.1% for prediabetes in Chinese adults [1]. Type 2 diabetes is the predominant form of diabetes in adults. Insulin resistance is frequently caused by overweight or obesity, which have been recognized as important risk factors for type 2 diabetes [2].

In the human body, vitamin D is mainly generated by cutaneous synthesis in response to UV radiation [3] and is then converted to 25-hydroxyvitamin D in the liver through hydroxylation; this form is the biochemical marker representing the vitamin D status in the human body [4]. Finally, the active form of vitamin D (1,25(OH)_2_D) is formed by 1-*α*-hydroxylase in the kidneys. Previously, vitamin D was considered to play a vital role in calcium homeostasis and bone metabolism. Recent studies indicated an association between low serum levels of 25(OH)D and cardiovascular diseases, diabetes, obesity, hypertension, and dyslipidemia [5]. However, the relationship between 25(OH)D and insulin resistance was debated [6–10]. Some studies also showed lower serum 25(OH)D concentrations in overweight and obese patients [11–13]. This inconsistency might be related to the body mass index (BMI). Furthermore, the relationship is still unknown in the Chinese population with different BMI.

In this study, we carried out a large clinical investigation (SPECT-China) to analyze the sex-specific differences of the relationship between the serum vitamin D concentrations and insulin resistance in the Chinese population.

## 2. Materials and Methods

### 2.1. Study Population

The SPECT-China study was a population-based cross-sectional survey of the prevalence of metabolic diseases and risk factors in eastern China. This study was performed in three rural sites in Zhejiang province, one urban and six rural sites in Jiangxi province, and three urban and three rural sites in Shanghai. Adults who had lived at their current residence for 6 or more months were recruited for our study from February to June 2014. The overall response rate was 90.8%. The selection and exclusion criteria were described previously [14]. Finally, 6597 subjects were included. This study was approved by the ethics committee of Shanghai Ninth People's Hospital affiliated to Shanghai Jiaotong University School of Medicine. Written consent was obtained from all participants, and the methods were carried out in accordance with the approved guidelines. A preliminary version of the work was presented at ENDO 2016 [15].

### 2.2. Examination of Indexes

Blood samples were collected from all participants after fasting for more than 8 hours and immediately centrifuged (2000 rpm for 15 min) at room temperature. Then, the samples were sent to a central laboratory certified by the College of American Pathologists. Biochemical indexes such as Cr, LDL, TG, HDL, and TC were measured using a BECKMAN COULTER AU 680 with the original kit. HbA1c and insulin were evaluated using an MQ-2000PT (Medconn Technology, Shanghai, China) and Abbott i2000 SR, respectively. Serum 25(OH)D was measured using a Siemens ADVIA Centaur XP with the original kit. The intra- and inter-assay coefficients of variance were 6.25% and 8.33%, respectively.

### 2.3. Definition and Classification

Diabetes, prediabetes, and normal glucose tolerance (NGT) were defined as previously reported [14]. Insulin resistance was evaluated using homeostasis model assessment of insulin resistance (HOMA-IR), which was calculated as the fasting glucose (mmol/L) × fasting insulin (mIU/L)/22.5 [16]. Body mass index (BMI) was calculated as weight (kg)/height squared (m^2^). Overweight was defined as 25 ≤ BMI < 30 kg/m^2^. Obesity was defined as BMI ≥ 30 kg/m^2^. The blood pressure and heart rate were measured three times using a sphygmomanometer (TERUMO-Elemano). The mean of the three measurements was used in the analysis. Waist circumference (WC) was measured 1 cm above the umbilicus. Demographic information and lifestyle risk factors were gathered from standard questionnaires by a trained staff. Drinking and smoking status were divided into never drinking/smoking and past or current drinking/smoking.

### 2.4. Statistical Analysis

Statistical analysis was performed using SAS9.4 for Windows (SAS Institute, Cary, NC, USA). Continuous variables were expressed as the mean ± SD. Because the HOMA-IR values were not normally distributed, we used a logarithmic transformation (base 10) to normalize the variable. Comparisons between groups were tested by analysis of variance (ANOVA) for the normally distributed variables. Pearson's correlation was used to analyze the relationship between 25(OH)D and other indexes. Three models were constructed in regression analysis. Model 1 was unadjusted. Model 2 was adjusted for age, smoking, and drinking. Model 3 was additionally adjusted for systolic blood pressure and BMI. All analyses were two-sided, and *P* < 0.05 was considered significant.

## 3. Results

### 3.1. Comparison between Different Groups

Serum 25(OH)D was categorized into quartiles, and the different indexes were compared between quartiles. Male subjects with higher 25(OH)D had reduced fasting insulin, HOMA-IR, LDL, TG, TC and elevated age, Cr, and HDL. Other potential risk factors, such as HbA1c, fasting glucose, WC, and BMI, showed no graded changes as 25(OH)D increased (Table 1). However, female subjects with higher 25(OH)D displayed reduced fasting glucose, fasting insulin, HOMA-IR, TG and increased age, HbA1c, Cr, LDL, and WC. HDL, TC, and BMI showed no graded changes as 25(OH)D increased (Table 2).

### 3.2. Correlation of Serum 25(OH)D with Other Parameters

The serum 25(OH)D values were inversely associated with HOMA-IR in male subjects (*r* = −0.055, *P* = 0.028) but not in female subjects (*r* = −0.005, *P* = 0.798). Both age TG and LDL were associated with vitamin D in males and females. Fasting insulin, BMI, TC, and HDL were associated with 25(OH)D in males. HbA1c and Cr was associated with 25(OH)D in females (Table 3).

### 3.3. Regression Analysis of 25-Hydroxyvitamin D and Insulin Resistance

The analysis of the total population showed that serum 25(OH)D concentration was significantly associated with log_10_ HOMA-IR in all three models. In males, serum 25(OH)D concentration was significantly associated with log_10_ HOMA-IR only in the overweight group, with unstandardized coefficients −3.39, −2.94, and −2.68 in three different models. However, in females, this association does not exist in all three different BMI groups (Table 4).

## 4. Discussion

The relationship between vitamin D and insulin resistance had been widely reported [7, 17, 18]. In our previous study, 25(OH)D was associated with HOMA-IR in a general Chinese population. In subgroup analysis, 25(OH)D was associated with HOMA-IR particularly in the overweight or prediabetes population [14]. In this study, we explored the relationship between 25(OH)D and insulin resistance in males and females, respectively. Additionally, the vitamin D concentration is higher in males than females. Thus, the relationship between 25(OH)D and insulin resistance might be different.

Previous studies showed lower 25(OH)D concentrations in overweight and obese patients, which might be caused by the storage of vitamin D in adipose tissue and decreased sunlight exposure in obese subjects [19, 20]. Wright et al. found that the plasma 25(OH)D was related to insulin resistance in overweight and obese adults [21]. El-Hajj et al. found that vitamin D supplementation did not improve the HOMA-IR in 257 elderly overweight individuals [22]. Jamka et al. performed a meta-analysis of overweight and obese subjects. The result indicated that vitamin D supplementation had no significant effect on the HOMA-IR index when considering the dose, time of vitamin D supplementation, and baseline vitamin D concentration in serum [10]. Furthermore, the relationship between vitamin D and insulin resistance is still uncertain in the overweight or obese population in China. Oral vitamin D supplementation reduced the hypersecretion of parathyroid hormone and insulin resistance in obese Chinese males [23]. However, correction of hypovitaminosis D did not improve the body mass index, plasma glucose, or lipid profile in subjects with metabolic syndrome [24]. Therefore, these analyses did not consider gender differences. These studies also included both male and female subjects, which may affect the results.

Our findings revealed that the association between vitamin D and HOMA-IR was different in males and females. We found a sexual dimorphism for the association between 25(OH)D with HOMA-IR. Moore et al. found an association between plasma 25(OH)D and HOMA-IR in males but not in females [25]. The subjects in this study were healthy young adults. The average BMI in males and females was 26.8 kg/m^2^ and 26.0 kg/m^2^, respectively, which is similar to the BMI values in our study (26.95 and 26.90, resp.). Furthermore, Wang et al. [26] indicated 25(OH)D is inversely related to HOMA-IR in young Chinese obese men with NGT. There was also disagreement of our study. Vigna et al. [27] pointed out no gender difference of vitamin D. However, they included a relatively small sample size with 385 patients who were all overweight or fat subjects.

The underlying mechanism of this relationship is unknown. Estrogen, an important regulator of energy metabolism, might play an important role in this relationship. Estrogen deficiency causes insulin resistance in menopausal women [28]. However, in postmenopausal women, estrogen-based replacement therapies improve insulin sensitivity and strongly reduce the incidence of type 2 diabetes [29]. In a bilateral ovariectomy mice model, estrogen treatment may reverse aspects of pathway-selective insulin resistance [30].

We also found that 25(OH)D was negatively associated with TG, TC, and LDL in males, but it was negatively associated with only TG in females. There was also uniform agreement on the negative relationship between 25(OH)D and TG [31]. The mechanism might be a suppression of the PTH secretion, which has been reported to reduce lipolysis in vitro [32]. Furthermore, vitamin D could increase the calcium level, which subsequently reduces hepatic TG formation and/or secretion [33, 34]. However, the effect of vitamin D supplementation on serum lipids is still uncertain [31].

Our study also had several limitations. The cross-sectional nature of the study means causality, and the direction of the associations observed cannot be determined. The study lacks gold standard methods for measuring insulin resistance and 25(OH)D: HOMA-IR is an indirect proxy measure of insulin resistance and is not the most reliable in accurately capturing insulin resistance. Serum 25(OH)D concentrations were measured over a span of five months in winter and spring by Centaur assay instead of liquid chromatography tandem-mass spectrometry (LC-MS/MS).

Use of BMI as a measure of obesity is limited as it does not account for lean/fat mass and other methods such as dual X-ray absorptiometry would have been preferable since percentage body fat is more closely related to both vitamin D levels and insulin resistance. We did not test related indexes, such as 2-hour OGTT, PTH, and calcium. Some confounding factors such as physical activity, sun exposure, and season of blood collection were unadjusted. Furthermore, this study did not obtain long-term follow-up results.

## 5. Conclusions

We found an inverse association between 25(OH)D and HOMA-IR in the overweight male Chinese population. This relationship might exert a protective effect against insulin resistance. Further prospective studies of vitamin D supplementation are still needed, especially in overweight males.

## Figures and Tables

**Table 1:** Comparison between different 25(OH)D subgroups in males.

Males	25OHD				
	Q1 (≤35.08)	Q2 (35.09–41.08)	Q3 (41.09–48.38)	Q4 (>48.38)	*P* for trend
N	704	703	704	702	
Age (year)	49.34 ± 13.78	50.29 ± 12.51	53.10 ± 12.79	57.92 ± 13.20	<0.001
HbA1c (%)	5.47 ± 10.1	5.38 ± 0.91	5.44 ± 0.86	5.47 ± 0.82	0.781
Fasting glucose (mmol/L)	5.67 ± 1.50	5.58 ± 1.33	5.67 ± 1.23	5.68 ± 1.29	0.695
Fasting insulin (pmol/L)	41.90 ± 48.75	39.82 ± 35.67	37.35 ± 32.59	33.98 ± 38.24	<0.001
HOMA-IR	1.60 ± 2.37	1.46 ± 1.54	1.40 ± 1.49	1.28 ± 1.74	<0.001
Cr (umol/L)	86.17 ± 15.51	87.48 ± 15.54	88.46 ± 20.74	87.93 ± 16.34	0.020
LDL (mmol/L)	2.97 ± 0.75	3.05 ± 0.72	2.90 ± 0.66	2.80 ± 0.65	<0.001
TG (mmol/L)	2.38 ± 2.88	2.05 ± 1.79	1.79 ± 1.38	1.39 ± 0.92	<0.001
HDL (mmol/L)	1.36 ± 0.33	1.35 ± 0.29	1.35 ± 0.30	1.42 ± 0.33	0.004
TC (mmol/L)	5.19 ± 1.10	5.18 ± 1.03	4.98 ± 0.91	4.91 ± 0.87	<0.001
WC (cm)	82.05 ± 9.24	83.28 ± 9.86	82.94 ± 8.97	82.41 ± 9.00	0.417
BMI (kg/m^2^)	24.31 ± 3.30	24.90 ± 3.80	24.69 ± 3.63	24.21 ± 3.24	0.903

HOMA-IR: homeostasis model assessment of insulin resistance; Cr: creatinine; LDL: low-density lipoprotein; TG: triglyceride; HDL: high-density lipoprotein; TC: total cholesterol; WC: waist circumference; BMI: body mass index. Data are shown as means ± SD.

**Table 2:** Comparison between different 25(OH)D subgroups in females.

Females	25OHD				
	Q1 (≤32.755)	Q2 (32.756–38.020)	Q3 (38.021–44.530)	Q4 (>44.531)	*P* for trend
N	946	947	947	944	
Age (year)	51.31 ± 14.03	51.27 ± 12.80	51.13 ± 12.97	55.59 ± 13.48	<0.001
HbA1c (%)	5.21 ± 0.85	5.27 ± 0.68	5.30 ± 0.76	5.39 ± 0.71	<0.001
Fasting glucose (mmol/L)	5.64 ± 1.18	5.58 ± 1.15	5.58 ± 1.32	5.54 ± 1.06	0.035
Fasting insulin (pmol/L)	43.29 ± 35.37	39.73 ± 25.88	40.94 ± 29.68	39.78 ± 27.09	0.036
HOMA-IR	1.63 ± 1.90	1.46 ± 1.21	1.53 ± 1.51	1.44 ± 1.23	0.015
Cr (umol/L)	66.83 ± 10.51	68.67 ± 11.49	69.94 ± 15.45	70.31 ± 11.43	<0.001
LDL (mmol/L)	2.84 ± 0.71	2.94 ± 0.78	2.94 ± 0.72	2.98 ± 0.73	0.001
TG (mmol/L)	1.59 ± 1.39	1.54 ± 1.70	1.40 ± 0.99	1.37 ± 0.78	<0.001
HDL (mmol/L)	1.52 ± 0.32	1.52 ± 0.31	1.53 ± 0.31	1.53 ± 0.31	0.332
TC (mmol/L)	5.06 ± 1.01	5.13 ± 1.04	5.09 ± 1.02	5.16 ± 1.05	0.251
WC (cm)	75.27 ± 9.94	76.11 ± 9.87	76.02 ± 9.48	77.78 ± 9.49	<0.001
BMI (kg/m^2^)	23.67 ± 3.70	23.87 ± 3.57	23.93 ± 3.66	24.02 ± 3.42	0.075

HOMA-IR: homeostasis model assessment of insulin resistance; Cr: creatinine; LDL: low-density lipoprotein; TG: triglyceride; HDL: high-density lipoprotein; TC: total cholesterol; WC: waist circumference; BMI: body mass index. Data are shown as means ± SD.

**Table 3:** Correlation of vitamin D with other parameters by sex.

Variable	Male		Female	
	*r*	*P*	*r*	*P*
Age (year)	0.265	<0.001	0.124	<0.001
HbA1c (%)	0.016	0.404	0.085	<0.001
Log HOMA-IR	−0.055	0.028	−0.005	0.798
BMI (kg/m^2^)	−0.049	0.009	0.026	0.109
Insulin (pmol/L)	−0.089	<0.001	−0.029	0.070
Fasting glucose (mmol/L)	0.001	0.942	−0.022	0.172
TC (mmol/L)	−0.124	<0.001	0.026	0.115
HDL (mmol/L)	0.088	<0.001	0.019	0.240
TG (mmol/L)	−0.194	<0.001	−0.068	<0.001
LDL (mmol/L)	−0.123	<0.001	0.057	<0.001
Cr (umol/L)	0.020	0.292	0.106	<0.001

HOMA-IR: homeostasis model assessment of insulin resistance; Cr: creatinine; LDL: low-density lipoprotein; TG: triglyceride; HDL: high-density lipoprotein; TC: total cholesterol; WC: waist circumference; BMI: body mass index. Data are shown as means ± SD.

**Table 4:** Association of 25(OH)D with insulin resistance in males and females, respectively.

	25(OH)D (nmol/l)		
	Model 1	Model 2	Model 3
Total	−2.07 (−2.95, −1.20)	−1.71 (−2.56, −0.85)	−1.45 (−2.38, −0.52)
Male			
BMI < 25 kg/m^2^	−1.43 (−3.50, 0.64)	−0.55 (−2.55, 1.44)	−0.37 (−2.44, 1.71)
25 ≤ BMI < 30 kg/m^2^	−3.39 (−5.57, −1.22)	−2.94 (−5.09, −0.79)	−2.68 (−4.96, −0.50)
BMI ≥ 30 kg/m^2^	−4.96 (−10.30, 0.38)	−2.98 (−8.37, 2.42)	−2.21 (−7.60, 3.18)
Female			
BMI< 25 kg/m^2^	−0.14 (−1.65, 1.37)	−0.45 (−1.95, 1.05)	−0.75 (−2.29, 0.78)
25 ≤ BMI < 30 kg/m^2^	−1.82 (−3.95, 0.30)	−1.94 (−4.05, 0.17)	−1.89 (−4.04, 0.26)
BMI ≥ 30 kg/m^2^	−2.91 (−7.23, 1.41)	−3.12 (−7.50, 1.25)	−3.20 (−7.60, 1.21)

Data are unstandardized coefficients (95% confidence interval). Model 1 unadjusted; model 2 adjusted for sex (in total subjects), age, smoking, and drinking; and model 3 adjusted terms for model 2, systolic blood pressure, and BMI.
